# Nutritional quality and consumer health perception of online delivery food in the context of China

**DOI:** 10.1186/s12889-022-14593-9

**Published:** 2022-11-19

**Authors:** Xiaoting Dai, Linhai Wu, Wuyang Hu

**Affiliations:** 1 School of Business, Jiangnan University, Wuxi, 214122 China; 2 Institute for Food Safety Risk Management, Jiangnan University, Wuxi, 214122 China; 3 Department of Agricultural, Environmental, and Development Economics, The Ohio State University, Columbus, 43210 USA

**Keywords:** Dietary health, Food environment, Health perception, Nutritional quality, Online food delivery

## Abstract

**Background:**

Today, the popularization of mobile internet technology has enabled the public’s need for food convenience and diversity arising from modern fast-paced lifestyles to be met at a relatively low cost. The digital age of the restaurant industry has arrived. Online food delivery (OFD) is rapidly developing globally. However, the public’s awareness of the nutritional quality of food through OFD and their knowledge of dietary nutrition remain to be investigated.

**Methods:**

In the context of China, this study attempts to evaluate the nutritional quality of best-selling OFD set meals (i.e., meal combos) based on the current official Chinese dietary guidelines 2022. It accomplishes this by collecting data on popular OFD restaurants among consumers in 115 Chinese universities from the restaurants’ delivery addresses. Moreover, 20,430 valid questionnaires were collected online from undergraduates, graduate students, and other young groups aged 18–30 throughout China for descriptive analysis to investigate consumers’ perceptions of the nutritional quality of food through OFD and its health impact.

**Results:**

The results of the nutritional quality evaluation of the OFD set meals ranged widely from 15 to 85, with a mean of 36.57 out of a possible maximum score of 100; and 89.56% scored less than 50. The nutritional quality of OFD foods was thus generally low. The nutritional quality of foods was negatively correlated with their popularity among consumers.

**Conclusions:**

Young OFD consumers generally paid low attention to dietary nutrition knowledge and seldom paid attention to nutritional quality when choosing OFD foods while the nutritional quality of OFD foods was generally low. Respondents subjectively reported that long-term consumption of OFD food caused weight gain, increased blood lipids, and gastrointestinal discomfort. They thought that the reason might be excessive oil, salt, and sugar in the food, while ignoring the balance between different types of food.

**Supplementary Information:**

The online version contains supplementary material available at 10.1186/s12889-022-14593-9.

## Background

The rapid development of the new “Internet Plus” economic form and the increase in broadband penetration has promoted the continuous expansion of electronic transactions across the world [1–3]. Online services are gaining popularity due to the convenience of electronic transactions, the wide range of suppliers, and the expansion of delivery services [4, 5]. As a new form of food consumption that is rapidly developing worldwide, online food delivery (OFD) provides consumers with online food ordering and delivery services, thereby changing traditional food production and retail practices. While solving the problem of time cost for individuals and families in food acquisition and preparation, OFD has also gradually changed consumer dietary consumption patterns. In particular, OFD has become more popular in the midst of the COVID-19 pandemic [6–9]. For example, although the size of the restaurant industry in China did not change significantly from 2019 to 2021, the size of OFD increased by 61.62% in the same time frame. In addition, the penetration rate of OFD increased from 3.87% in 2015 to 19.92% in 2021 [10]. In 2021, there were 544 million OFD users in China [3], and more than 40% of restaurants in China provide both online and in-person food services [11].

The global OFD market will reach USD 339.3 billion by 2022, and it is further estimated that the global OFD market will see its average annual growth rate remain at 8.28% from 2022 to 2026 [12]. The rise of OFD has greatly changed the food environment,^1^ triggering changes in the practices of food production, transportation, and consumption worldwide. Consumers are increasingly buying food through online platforms, which has partially replaced traditional home cooking or dine-in patterns.^2^

However, OFD may have negative effects. For example, by saving time spent on food shopping and home cooking, OFD may also reduce physical activity time, resulting in an increase in health problems associated with sedentary lifestyle [8, 13, 14]. Moreover, due to lack of information or labeling, it is difficult to guarantee the nutritional quality of OFD food [15–17], not to mention meeting the individualized nutritional needs [18, 19]. These may lead to a negative impact on public health. The increasing prevalence of chronic diseases in younger age groups due to dietary and nutritional health problems has become a widespread social problem in countries including China [20–23]. Therefore, it is of essential importance to investigate the nutritional value of OFD food and its impact on public health, especially on the risk of chronic diseases. However, only very few reports exist on this topic to date. It is of particular relevance to study this topic in China given the rapid development and huge market size of its OFD market.

According to Statista [12], the global OFD market will reach USD 339.3 billion by 2022, while China alone will account for USD 158.1 billion, equivalent to 46.60% of the global market. In other words, China has the largest OFD market, the greatest young consumer groups, and also the largest group of undergraduates and graduate students in the world. It is the uniqueness that other countries may not have. Moreover, due to the uncertainty of the COVID-19 pandemic, and more importantly, the convenience and relatively low prices of food ordered online, it is foreseeable that the online food delivery industry will develop faster in China in the future. In this sense, it is unique, forward-looking and representative to study the nutritional quality of OFD foods in China. In general, in addition to the special OFD foods for special groups, the OFD foods in western developed countries are also standardized produced and processed as in China. Objectively, the nutrition of OFD foods is difficult to effectively meet the health needs of the most consumer groups. Therefore, this study is representative to some extent, and the research conclusions have certain reference value for other countries. Nevertheless, the conclusions of this study are merited of greater reference value to China due to the differences in dietary structure and culture among countries in the world.

In the context of OFD’s rapid development and high penetration rate in China, this study aims to evaluate the nutritional quality of popular OFD foods. We analyze the best-selling OFD set meals of the 345 most popular OFD restaurants delivering to addresses near 115 different universities across China. Moreover, a survey was conducted among undergraduates, graduate students, and other young groups aged 18–30 to investigate consumers’ perception of the low nutritional quality of OFD food and analyze its health impact.

### Literature review

In the last few decades, rapid economic development and the need for convenience have led to a rapid global rise of food away from home (FAFH),^3^ including dining out, takeaways, OFD, and other ways of preparing or consuming food outside of home [24]. This has raised concerns regarding the relationship between FAFH quality and public health. Lachat et al. (2012) [25] and Wallard-Cole et al. (2021) [24] concluded that FAFH led to increased intakes of energy, total fat, saturated fat, and sodium, as well as decreased micronutrient intake among consumers, and argued that the nutritional quality of FAFH generally did not meet the daily nutritional needs of consumers.

OFD is one of the most important ways through which FAFH has developed rapidly worldwide. Both the size of OFD users and the scope of influence OFD has on the food landscape have attracted the attention of scholars. Young people are the main users of OFD globally [26]. In the US, Canada, and France, consumers aged 18–34 make up more than one-third of all OFD consumers [12]. In Australia and New Zealand, more than 25% of OFD consumers are young people aged 15–34 [27]. Examining a Malaysian context, Eu and Sameeha (2021) [28] reported that OFD consumers were mainly college students aged 19–29. In China, more than 50% of OFD consumers are young people aged 18–30 [29]. Moreover, a survey on OFD conducted in China showed that 90.48% of the students had used OFD services and 92% of the restaurants around the university had joined online platforms designed to facilitate OFD [30].

Meanwhile, the impact of OFD on health has attracted great interest from scholars. The overall conclusion is that food through OFD cannot meet individuals’ nutritional needs. For example, Horta et al. (2021) [31] investigated online platforms where OFD is hosted and found that most restaurants provided a large number of pre-made foods and beverages without sufficient customization to fit individual needs. Partridge et al. (2020) [5] and Brar and Minaker (2021) [18] also found that most OFD foods popular with consumers were from takeout franchises with standardized production, which did not include much nutritional customization. Likewise, Stephens et al. (2020) [14] suggested that standardized online ordering of fast food similar to pizza was popular among OFD consumers in the US. Horta et al. (2022) [19] found that traditional meals and pasta set meals^4^ were the food items most frequently pushed to consumers by Brazilian OFD platforms.

In order to further verify the performance that the nutrition of OFD foods is difficult to effectively meet people’s health needs, scholars have conducted a series of studies on this. For example, the “Ghost kitchens^5^” style production and processing mode [32], resulting in the increasing and serious public health problems [4]. Zang et al. (2018) [33] found that the low nutritional quality of FAFH was manifested in increased intake of energy, fat, and carbohydrates by consumers. Goffe (2020) [15] pointed out that the convenient meals available via OFD ordering platforms popular with the global public were generally characterized by high energy and low nutritional quality. Based on data from three countries, Poelman et al. (2020) [16] suggested that the majority of foods through OFD were unhealthy, and that consumers living in communities with lower socioeconomic levels only had access to a smaller proportion of healthy food types. Partridge et al. (2020) [5] used data from two international cities to evaluate the characteristics and nutritional quality of foods on OFD platforms, concluding that the most popular foods on the platforms were unhealthy. Brar and Minaker (2021) [18] also reported that foods available on OFD platforms in Canada were of low nutritional quality and did not meet the requirements of healthy dietary guidelines. After assessing the nutritional quality and marketing attributes of food on Australian OFD platforms, Wang et al. (2021) [17] speculated that OFD platforms promote unhealthy food, and strongly suggested scholars to further conduct specific research on the nutrition of OFD foods. Similarly, Keeble et al. (2020) [34] emphasized the necessity to investigate how OFD affects dietary patterns and public health in their research.

Moreover, some studies have investigated specific health problems caused by changes in the food environment as a result of OFD. For example, FAFH, which is closely supported by OFD, has been shown to be generally high in calories, added sugar, saturated fat, salt, and low in nutritional value [26, 35–38]. These food characteristics have been proven as key risk factors for chronic diseases such as obesity, high cholesterol, diabetes, and hypertension [20–23]. Nago et al. (2014) [39] and Wellard-Cole et al. (2018) [40] also reported a positive correlation between the degree of weight gain and the frequency of FAFH consumption. McCrory et al. (2019) [41] believed that the rising obesity rate is inextricably linked to the popularity of FAFH. Moreover, Janssen et al. (2018) [42] and Dana et al. (2021) [43] suggested that the low-nutrient food through OFD is a key factor leading to overweight and obesity. Stephens et al. (2020) [14] and Horta et al. (2022) [19] pointed out that long-term reliance on OFD may lead to chronic diseases such as obesity, hypertension, and diabetes.

Numerous studies have discussed the public health issues caused by food through OFD. However, Stephens et al. (2020) [14], Partridge et al. (2020) [5], and Keeble et al. (2020) [34] pointed out that despite the rapid increase in public dependence on OFD, few objectives and generally accepted research exist demonstrating the health impact of food through OFD from either individual or public health perspectives. Research on the nutritional quality of meals that focuses on consumers in the context of Chinese OFD platforms is even more scarce despite the fact that China has the largest OFD market in the world.

This paper attempts to fill the gaps in the above literature. Considering that the main users of OFD in China are young people [29], the best-selling OFD set meals from 345 most popular OFD restaurants delivering to consumers on or near 115 different university campuses in China were used as our sample. Different from previous studies, the efforts and contributions of this paper are as follows: Based on the background of rapid development, large scale, large number of users and high penetration rate of OFD in China, we evaluate the nutritional quality of OFD foods based on the dietary guidelines published by the Chinese Nutrition Society, which is believed to be the first study in this field. Moreover, an online survey was conducted among undergraduates, graduate students, and other young groups aged 18–30 to investigate consumers’ perception of the nutritional quality of OFD food and summarize its health impact, which is first to use China as a case.

## Methods

### Data collection and nutritional quality assessment of OFD food

#### Selection of OFD platforms

One objective of this study is to evaluate the nutritional quality of foods available on Chinese OFD platforms. Food on the “Meituan” platform was used to evaluate the nutritional quality of OFD food. Founded in 2010, Meituan is one of the two OFD titans in China. According to the *2020–2021 Research Report on Food Delivery Industry Development in China* [10] and *Meituan Annual Financial Statements (2017–2021)* [44] and as shown in Table 1, Meituan’s share of China’s OFD market increased from 62.42% in 2017 to 75.17% in 2021, with an average annual increase of 3.17%.

**Table 1:** OFD market size in China, and Meituan’s market share (100 million yuan, %)

Year	Online food delivery market size	Online food delivery transaction volume of Meituan	Market share of Meituan
2017	2174	1711	62.42%
2018	4250	2828	66.54%
2019	5779	3927	67.95%
2020	6646	4889	73.56%
2021	9340	7021	75.17%

#### Selection of OFD ordering locations and foods

Based on the findings from previous studies on main OFD consumer groups, young people aged 18–30, mainly students, are chosen as respondents for the study. A total of 115 Chinese universities, including Peking University, Tsinghua University, University of Science and Technology of China, and Fudan University, were selected as our data collection basis. The top three restaurants with the highest OFD monthly sales (each received over 10,000 orders) to each university were selected resulting in a total of 345 restaurants. The best-selling set meal from each selected restaurant was used as a sample for nutritional quality evaluation.

To ensure data integrity and consistency, and considering the usual meal time among Chinese consumers, all data were collected from the 345 restaurants including only lunch time (11:30–13:00) and dinner time (17:30–18:30) from May 01 to June 30, 2022. These best-selling set meals of the selected restaurants were sorted for types and weight and assessed for nutritional quality.

#### Nutritional quality evaluation method

Every country or region has different dietary nutrition standards. For example, a total of 20 countries or regions have published nutrient recommendations for milk and dairy products, and the recommended values in Asian countries are generally lower than those in Europe, America, and Oceania [45]. Unlike studies in other countries that evaluated the nutritional quality of OFD food [18, 46], the evaluation in this study is based on the *Dietary Guidelines for Chinese Residents (2022)* (referred to as the Guidelines hereafter). The Guidelines were developed by the Chinese Nutrition Society after drawing on the dietary guidelines in other countries and translating the existing evidence on dietary nutrition into food-based dietary guidelines based on the reality of China. The goal is to help individuals maintain health and reduce the incidence of nutrition-related diseases. They are more in line with the characteristics and changing trends of Chinese citizens’ dietary structure and the reality of food production and supply in China. Using the Chinese Guidelines can avoid the bias caused by using the nutritional quality standards of other countries.

According to the Guidelines, the 12 food items that Chinese people need on a daily basis and their daily recommended intake are shown in Table 2. Following the scoring methods used by Reedy et al. (2018) [47] and Bar and Minaker (2021) [18] for the US Healthy Eating Index-2015 (HEI-2015), a maximum total score of 100 was established for a perfect balance of the 12 food items and their recommended daily intake (Table 2). Food combinations were scored accordingly. A higher total score indicates a healthier food combination. Healthier food combinations mean better balanced meals containing items from various categories. The higher the content of a desirable food item in the set meal, the higher the component score. However, the opposite is true for some other items such as sodium and cooking oil; in other words, the lower the content of these items, the higher the overall score.

**Table 2:** Dietary nutrition evaluation criteria

Food items (recommended daily serving size)	Description	Maximum score	Criteria for maximum score
Fruits (200–350 g)	Apples, pears, bananas, grapes, and pineapples, etc	10	≥ 83 g
Vegetables (300–500 g)	Celery, carrots, cabbage, spinach, and eggplant, etc	10	≥ 100 g
Milk and dairy products (300–500 g)	Milk, yogurt, and cheese, etc	10	≥ 100 g
Poultry and meat (40–75 g)	Pork, beef, lamb, chicken, and duck, etc	5	≥ 13 g
Aquatic products (40–75 g)	Fish and shrimp, etc	5	≥ 13 g
Cereals (200–300 g)	Rice, flour, wheat, corn, and buckwheat, etc	10	≥ 67 g
Whole grains and beans (50–150 g)	Barley, wheat, and rye, etc soybeans and red beans, etc	5	≥ 17 g
Potatoes (50–100 g)	Sweet potato, potato, yam, taro, and cassava, etc	10	≥ 17 g
Soybeans and nuts (25–35 g)	Soy milk, tofu, and dried tofu, etc.; almonds, pine nuts, and walnuts, etc	10	≥ 8 g
Eggs (40–50 g)	Chicken eggs and duck eggs, etc	5	≥ 13 g
Sodium (≤ 5 g)	Sodium	10	≤ 1667 mg
Edible oil (25–30 g)	Sum of all saturated, trans, monounsaturated, and polyunsaturated fats	10	≤ 10 g
Total score	N/A	100	N/A

All 12 food items were evaluated in grams (g) except sodium, which was evaluated in milligrams (mg). The content in column 4 “Criteria for maximum score” is the recommended daily intake per meal, which is converted from the recommended daily intake defined in the Guidelines. A score of 10 was assigned if the mass of a food item in the sample met or exceeded the “criteria for maximum score”; otherwise, it was scored according to the percentage. For example, if the fruit content of a sample set meal was 40 g, the score would be 4.82 (40/83*10)

The maximum scores for the 12 food items listed in Table 2 are not all the same. For example, the maximum score for poultry and meat (as a combined category) as well as aquatic products is 5, respectively, which are different from the maximum possible score of 10 for most other food items. There are two reasons for this. First, the Guidelines state that poultry, meat, and aquatic products are all animal products, and hence their nutritional components are complementary. Second, the maximum scores for total meats and seafood (as a combined category) and aquatic products set by HEI-2015 are also 5 for each category.

In addition, to evaluate nutritional quality, the following principles were used:

- If a food item is present in the menu without content description, based on the principle of conservative estimation, it is assumed that the criterion is met and a corresponding score is assigned (for example, if “a serving of vegetables” was stated in the menu, a score of 10 would be assigned to “vegetables”);
- If there is no set meal in the menu, the top three best-selling food items in the menu is selected and combined for nutritional quality evaluation;
- If the text description in the menu does not match the picture, the text description prevails;
- The contents of sodium and edible oil in the food are determined using Boohee.^6^ Specifically, the content of edible oil can be estimated by calculating the sum of saturated, trans, monounsaturated, and polyunsaturated fats. If the content of sodium or edible oil cannot be determined in the software, the median score for that item, i.e., 5, is assigned.

### Online survey

#### Questionnaire design

On the basis of previous qualitative research [9, 28, 46, 48], an online questionnaire was designed to evaluate current OFD consumption status, consumers’ perception of OFD food nutritional quality, and how OFD affects consumers’ health. The questionnaire contains two parts. The first part covers respondent demographics, such as gender, age, education, marital status, personal and family income, occupation, and the amount of OFD expenditure. The second part focuses on how respondents make OFD food choices, their nutritional and dietary needs, their perception of the nutritional quality of OFD food, and their perception of health after OFD food consumption. The detailed questionnaire contents are presented in the online supplementary material (see Additional file 1).

A small pilot test was first conducted to check the quality, accuracy, and readability of the questionnaire. The formal large-scale survey was conducted by creating an online link on a website and sharing the link via a professional marketing firm, which includes venues such as social media. Respondents’ informed consent was obtained on the first page of the questionnaire before commencement of data collection.

#### Sample demographics

The respondents of this paper must be the consumers with the experience of buying and consuming OFD food. According to this, and the questionnaires submitted by the respondents without such requirements are rejected. Table 3 shows the sample demographics of the survey. A total of 20,430 valid online questionnaires were collected from undergraduates, graduate students, and other young consumers aged 18–30. The majority of the respondents (87.37%) lived in cities of various sizes, which is consistent with the fact that OFD is more popular in urban than in rural areas in China [28, 43, 49]. Students, company employees, and public institution employees accounted for 34.51%, 30.25%, and 12.92% of the sample, respectively. Moreover, more than 80% of the respondents had a bachelor’s degree or above. In addition, 81.75% of the respondents had a personal pre-tax annual income of less than 150,000 yuan.

**Table 3:** Demographics of respondents

Group	Sample size (n)	Proportion (%)
**Gender**		
Male	8381	41.02
Female	12,049	58.98
**Age (year)**		
18–30	20,430	100.00
**Place of residence**		
Cities and towns	17,851	87.37
Countryside	1419	6.95
rural–urban continuum	1160	5.68
**Education**		
Junior high school or lower	659	3.23
High school	1170	5.73
Junior college	2091	10.23
Bachelor’s degree	9801	47.97
Master’s degree or higher	6709	32.84
**Personal annual income (yuan)**		
< 30,000	7249	35.49
30,000–50,000	2271	11.11
50,000–100,000	3941	19.29
100,000–150,000	3240	15.86
> 150,000	3729	18.25
**Occupation**		
Company employee	6181	30.25
Public institution employee	2639	12.92
Civil servant	983	4.85
Farmer	245	1.22
Self-employed/unemployed/retired	3320	16.25
Student/graduate student	7050	34.51
**Frequency of Purchasing OFD (per week)**		
1 time	5192	25.42
2 times	3168	15.51
3 times	4935	24.16
4 times or more	4182	20.47
never	2953	14.45

## Results

### Results of nutritional quality evaluation of OFD foods

The 345 OFD set meals comprised 143 rice set meals (41.45%), 51 porridge and pastry set meals (14.78%), 49 crayfish and barbecue set meals (14.20%), 38 fast hot pots set meals (11.01%), 25 rice noodles and wheaten food set meals (7.25%), 13 fried chicken and skewers set meals (3.77%), 13 salad set meals (3.77%), and 13 pizzas and hamburgers set meals (3.77%).

**Fig. 1:**
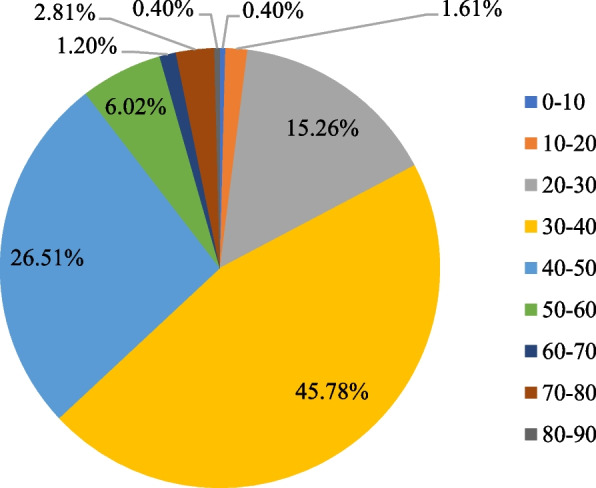
Distribution of nutritional quality scores of OFD foods (% in total sample)

The nutritional quality evaluation of three example OFD set meals is given in Table 4. Out of a maximum score of 100, the total score of the three OFD set meals ranged widely from 15 (crispy fried chicken set meal) to 85 (beef and chicken breast salad set meal), with a mean of 36.57 out of 100. In particular, as Fig. 1 displays, of all the set meals considered in this study, 65.05% scored below 40, and 89.56% scored below 50. It is thus clear that most OFD set meals have poor nutritional quality falling far below recommended values. They lacked fruit, milk and dairy products, aquatic products, whole grains and beans, soybeans and nuts, and eggs, and were high in sodium and cooking oils. The only food items with high scores were poultry and meat, and cereals.

**Table 4:** Nutritional quality of three example meals through OFD evaluated based on the Guidelines

Food item [maximum score]	OFD set meal examples			Mean score of 345 set meals
	**No.1**	**No.2**	**No.2**	
Fruits [10]	0	5	0	**0.27**
Vegetables [10]	10	10	10	**5.85**
Milk and dairy products [10]	0	0	0	**0.08**
Poultry and meat [5]	5	5	5	**4.61**
Aquatic products [5]	0	0	0	**0.75**
Cereals [10]	10	10	10	**9.60**
Whole grains and beans [5]	0	5	0	**0.21**
Potatoes [10]	0	0	0	**2.39**
Soybeans and nuts [10]	10	10	0	**1.77**
Eggs [5]	0	5	5	**1.10**
Sodium [10]	0	10	0	**4.27**
Edible oil [10]	10	10	5	**5.66**
**Total score**	**45**	**70**	**35**	**36.57**

**Fig. 2:**
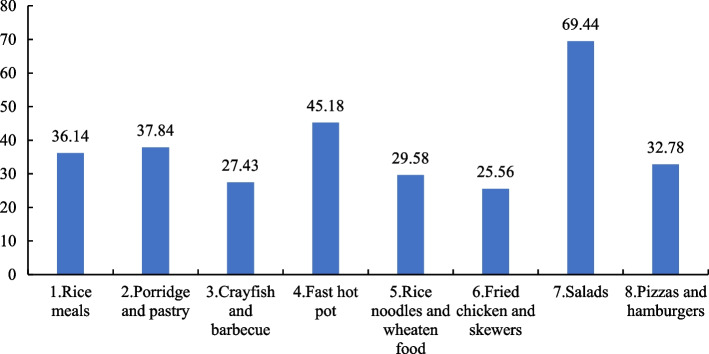
Nutritional quality scores of OFD meal types ranked by purchase frequency

Figure 2 shows the nutritional quality scores of different meals ranked according to frequency of purchase. In particular, “salads”, which ranked seventh in terms of frequency of purchase, scored the highest in nutritional quality. “Rice meals” and “porridge and pastry” ranked first and second in terms of purchase frequency, together accounting for more than 50% of the total sampled meals. However, both had a nutritional quality score of less than 40. This suggests that the OFD foods consumed by more than half of the consumers did not meet the Guidelines’ dietary nutritional recommendations.

### Online survey results

#### Consumer perception of nutritional quality

**Fig. 3:**
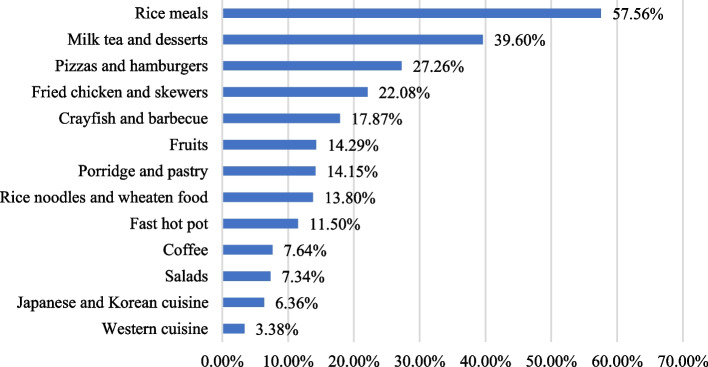
Proportion of popular OFD meal types based on online survey data (%)

Based on the online survey results, the popularity of the 13 OFD meal types is illustrated in Fig. 3. Obviously, “rice meals” were the most popular, accounting for 57.56% of the sample. This is in line with the data collected from the OFD platform reported in this study. However, as Fig. 2 suggests, the mean nutritional quality score of this meal type was only 36.14, which was 33.30 points lower than the highest score, which was earned by “salads” (Fig. 2). Because “milk tea and desserts,” which ranked second in popularity, are generally not considered a daily meal type, data were not collected for this category from the OFD platform. Therefore, the nutritional quality of “milk tea and desserts” was not evaluated in this study. However, according to Li and Yang (2017) [50] and Zheng (2021) [51], milk tea is generally an unhealthy food. The nutritional quality scores of “pizzas and hamburgers,” “fried chicken and skewers,” and “crayfish and barbecue,” which respectively ranked third, fourth, and fifth, had nutritional scores of 32.78, 29.58, and 27.43, far from meeting the Guidelines’ dietary nutritional recommendations. In contrast, “salads,” the meal type with the highest mean nutritional quality score shown in Fig. 2, had a very low purchase frequency of 7.34%. It ranked 11th in the list of 13 options in Fig. 3, which is consistent with the data collected from the OFD platform.

**Fig. 4:**
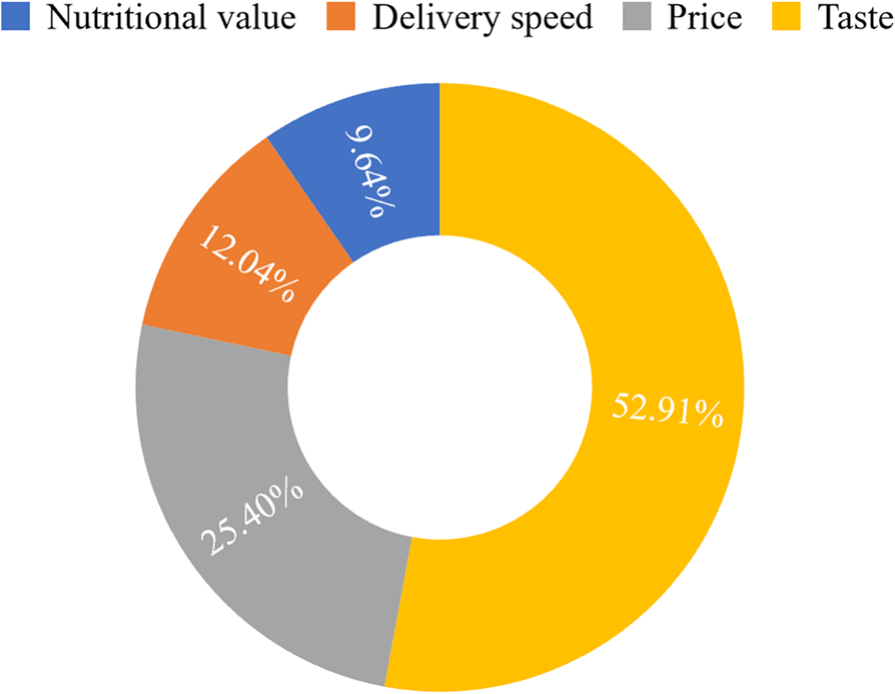
Concerns when ordering food online (%)

**Fig. 5:**
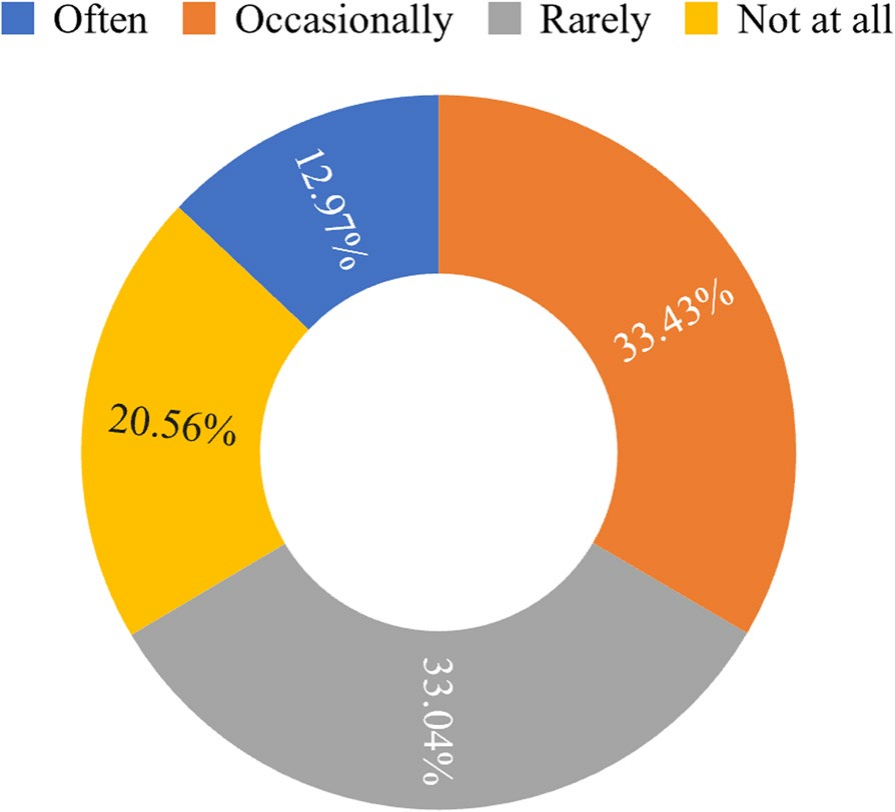
Level of attention to dietary nutrition knowledge (%)

As shown in Fig. 4, when making OFD food choices, 52.91% of the respondents paid most attention to taste, followed by price and delivery speed, and lastly nutritional value. This shows that consumers had low concern for dietary nutrition and health. In addition, respondents’ level of attention to dietary nutrition knowledge is shown in Fig. 5. Only 12.97% of respondents paid attention to this knowledge often and 33.43% occasionally paid attention. The majority either rarely or paid no attention at all. This result may indirectly indicate that the general public in China has not been too concerned about dietary nutrition. Young people in China have a low interest in acquiring, understanding, and using dietary nutrition knowledge related to their health. In addition, another possible reason for this result may be that young consumers tend to think they are overall healthy and thus care little about acquiring or using this knowledge.

#### Consumer health perceptions

**Fig. 6:**
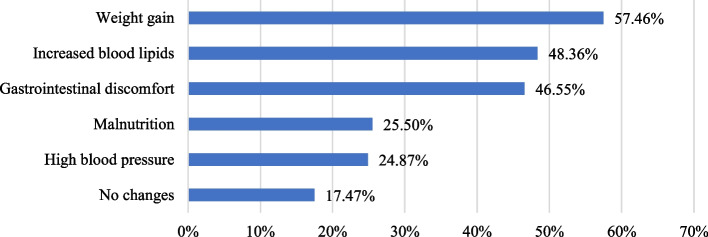
Perceived health changes after long-term consumption of OFD food (%)

The online questionnaire also included consumer self-evaluated physical health changes after long-term consumption of food through OFD. As shown in Fig. 6, 57.46% of the respondents believed that long-term consumption of food through OFD led to their weight gain. Nearly 50% reported increased blood lipids and gastrointestinal discomfort. Only 17.47% believed that long-term consumption of food through OFD had no effect on their physical health. Therefore, this study supports the notion that popular food ordered through OFD does not meet consumers’ daily nutritional needs. Long-term consumption of food through OFD may have a negative health impact, and should be included in the public discussion on how food through OFD may be related to chronic diseases, such as obesity, hyperlipidemia, hypertension, and type 2 diabetes.

**Fig. 7:**
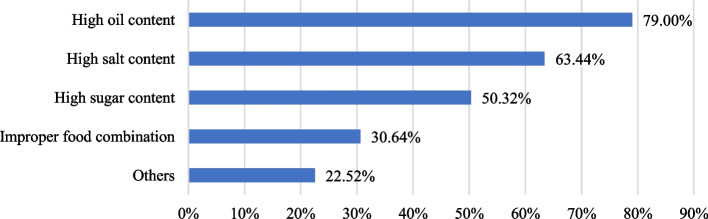
Reasons consumers believe that food through OFD does not meet health standards (%)

In addition, the respondents who believed that consuming food through OFD had an impact on their health were further asked about the signs and reasons of such a health impact (Fig. 7). The most reported reason was excessive cooking oil (79.00%), followed by high salt content (63.44%), high sugar content (50.32%), and improper balances of different types of food (30.64%). Obviously, the problem of high oil, salt, and sugar intake, which can lead to chronic diseases such as obesity, hyperlipidemia, and hypertension [52, 53], is of concern to consumers. It indicates that consumers are well-educated in this respect. However, the fact that of food ordered through OFD has improper nutrition balances has not been given adequate attention by young consumers.

#### Robustness test

In order to ensure the robustness of the cross-sectional study method and the representativeness of samples, this paper also collected data of OFD food around other types of ordering places. Similarly, using the *Dietary Guidelines for Chinese Residents (2022)* as the standard to evaluate the nutritional quality of OFD food by cross-sectional research method. The most popular shopping malls in 20 cities^7^ in China were selected as the OFD ordering places. The data of set meals from the top three restaurants with monthly sales volume around each shopping mall were selected as samples, that is, the set meals with the largest sales volume in 60 restaurants were selected as samples to evaluate the nutritional quality. The results show that the average score of nutritional quality assessment of the 60 online meals was 37.65 out of 100, among which 63.85% of the meals scored less than 40 out of 100, 89.05% of the meals scored less than 50 out of 100. The types of meals were also relatively single. It is not difficult to find that still be concluded that the nutritional quality of OFD food is generally low after selecting different types of places for ordering OFD food.

## Conclusions and policy implications

This study uses the best-selling set meals of the 345 most popular restaurants surrounding 115 universities in China as the basis to gauge the nutritional quality of food through OFD. The study uses data obtained from the super-leading OFD platform provider Meituan and creates the nutritional valuation based on China’s *Dietary Guidelines for Chinese Residents (2022)*. A follow-up online survey was administered among undergraduates, graduate students, and other young groups aged 18–30. The survey investigated consumer choices of food through OFD, as well as their perception of the nutritional quality and health impact of food ordered through OFD. We find that foods acquired through OFD generally had low nutritional quality scores. This is consistent with the findings of Bar and Minaker (2021) [18] regarding the nutritional quality of foods though OFD in North America. Meal types popular with young consumers had low nutritional quality, far from meeting the recommendations of the Guidelines. Relatedly, meal types with high nutritional quality were not common choices for young consumers. Furthermore, the vast majority of young consumers only paid attention to the taste and price of food, but ignored the nutritional value when using OFD. They generally had low awareness and might even lack basic appreciation of dietary nutrition and health. Weight gain, increased blood lipids, and gastrointestinal discomfort were the most common physical health changes perceived by young consumers after long-term consumption of food through OFD.

Our results may provide insights into improving the nutritional quality of food through OFD. The implications can be on both the supply side and the demand side [54]. Like in most countries in the world, a restaurant or food service usually does not make all their food items available for online ordering. For food providers, reducing the amount of unhealthy food and increasing the amount of healthy food offered through the online platform might be an essential component to improve the nutritional quality of food through OFD. However, if such an action reduces profit, food providers may not be willing to commit. This will need the effort from the demand side. Consumer education campaign can promote consumer awareness and knowledge about their nutritional health and reduce the intake of unhealthy or imbalanced food. As far as China is concerned, based on the fact that dietary nutrition and health knowledge has not received widespread attention from young consumers, efforts should be dedicated to this particular group to promote the *Dietary Guidelines for Chinese Residents (2022)*. Many individuals order food through OFD due to its convenience, thus, increasing the convenience of healthy food preparation for consumers might assist consumers making healthier choices. More drastic approaches that may encounter some level of pushbacks but can nevertheless be powerful include limiting the amount of unhealthy food by each customer per order.

Several areas of extension exit. First, an immediate venue is to apply our framework to the broader public to include consumers of all ages. Second, consumer heterogeneity such as age and gender may lead to large variation in their food ordering and nutritional health response to OFD. Further work to explore this heterogeneity and establish situational context of OFD is likely useful. Third, we used respondents’ self-reported health status to describe the impact of food through OFD. Additional investigation can consider tying food nutritional data with the amount and variety of ordered food through a consumer diary. Finally, one can apply the framework and test the general applicability of the conclusions in this study in a non-Chinese context.

## Supplementary Information

**Additional file 1.** Questionnaire contents.

## Data Availability

The datasets used during the current study are available from the corresponding author on reasonable request.

## Abbreviations

OFD: Online Food Delivery
FAFH: Food Away from Home
HEI-2015: Healthy Eating Index-2015
CNNIC: China Internet Network Information Center

## Footnotes

1 Food environment is defined as a collection of environmental, opportunity, physical, economic, policy, and sociocultural factors that affect food choices and individual nutritional status [55].

2 Penetration rate is the ratio of the target product or service to the product or service that the existing market may have, and is one of the methods to calculate the influence of a product or service. For example, Yeo and Kim (2019) [56] assessed the global smartphone penetration rate.

3 FAFH is defined as food prepared outside the home, and includes food that is eaten out, ordered in (taken out), and/or delivered [57].

4 In China, except liquid food and drinks, meal combos are often presented in a single container. As such, a meal combo is more commonly known as set meals in China.

5 Ghost kitchens, also known as cloud or dark kitchens, are restaurants that prepare meals solely for the delivery market, with no dine-in area or service staff [4].

6 Boohee is a popular App in China providing consumers with information such as nutrient calculation, calorie calculation, and diet analysis.

7 The top 20 cities in China were selected according to the 2022 City Business Charm Ranking released by CBN—New First-Tier Cities Research Institute (2022) [58]. Available at: https://view.inews.qq.com/wxn/20220608A049WY00?(2022–06-08).
