# Supplementary material for: Nutritional quality and consumer health perception of online delivery food in the context of China
Source: BMC Public Health. 2022 Nov 19;22:2132. doi: 10.1186/s12889-022-14593-9 (PMC9675956; doi:10.1186/s12889-022-14593-9)
Supplement: Supplementary file 1 — Additional file 1. Questionnaire contents. [file 12889_2022_14593_MOESM1_ESM.docx]

**Additional file 1:**

**Questionnaire contents**

| **Questions** | **Options** |
| --- | --- |
| Your gender | （1）Male；（2）Female |
| Your age | （1）18-30 years old；（2）others |
| Your education level | （1）Junior high school or lower；（2）High school；（3）Junior college；（4）Bachelor’s degree；（5）Master’s degree or higher |
| Personal annual income (yuan) | （1）less than 30,000；（2）30,000–50,000；（3）50,000–100,000；（4）100,000–150,000；（5）>150,000 |
| Your occupation | （1）Company employee；（2）public institution employee；（3）Civil servant；（4）Farmer；（5）Self-employed/unemployed/retired；（6）Student/graduate student |
| How often do you buy food and beverage through third-party delivery platforms such as “Meituan” and “Ele. Me” | （1）1 time per week；（2）2 times per week；（3）3 times per week；（4）4 times per week or more；（5）never |
| What types of online delivery food do you often buy (multiple choice, up to 3 items) | （1）Rice meals（2）Pizzas and hamburgers（3） Fast hot pot（4）Crayfish and barbecue（5）Fried chicken and skewers（6）Rice noodles and wheaten food（7）Porridge and pastry（8）Japanese and Korean cuisine（9）Western cuisine（10）Salads（11）Coffee（12）Fruits（13）Milk tea and desserts |
| What is your concern when ordering food online | （1）Price（2）Delivery speed（3）Taste（4）Nutritional value |
| How often do you make attention to dietary nutrition knowledge | （1）Not at all；（2）Rarely；（3）Occasionally；（4）Often |
| Do you think online delivery food can provide the daily required dietary nutrition | （1）Yes;（2）No |
| How do you feel about your health after you take online delivery food | （1）Healthier（2）Unhealthier（3）No changes |
| What kind of changes do you think your body will have after eating online delivery food for a long time? (Multiple choice) | （1）Weight gain（2）Increased blood lipids（3）Gastrointestinal discomfort（4）Malnutrition（5）High blood pressure（6）No changes |
| If you think online delivery food is unhealthy, what is the reason? (Multiple choice) | （1）Improper food combination（2）High oil content（3）High salt content（4）High sugar content（5）Other |
